# Trichobezoar as a cause of pediatric acute small bowel obstruction

**DOI:** 10.1002/ccr3.2576

**Published:** 2019-12-18

**Authors:** Allan Mun Fai Kwok

**Affiliations:** ^1^ North West Regional Hospital Burnie TAS Australia

**Keywords:** bezoar, pediatric, small bowel obstruction, trichobezoar, trichotillomania

## Abstract

This case serves to raise awareness of trichobezoar as a diagnosis in young children who present with abdominal pain, a palpable mass, and signs of acute small bowel obstruction.

## Graphical abstract

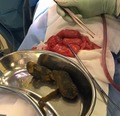

## 1 INTRODUCTION

Bezoars are hard indigestible masses which are most commonly encountered in the stomach because the pylorus acts as a natural impediment to further passage.^1^ The term is derived from the Arabic word “badzehr” meaning antidote, since bezoars from animals were traditionally used in ancient times as cures for poison and even prized as precious stones.^2^, ^3^ Several types of bezoar have been described including phytobezoar (plant material), trichobezoar (hair), lithobezoar (stones), pharmacobezoar (tablets), plasticobezoar (plastic material), and lactobezoar (milk proteins).^2^ Phytobezoars are the most common type, while trichobezoars account for less than 6% of all cases of bezoars found in humans.^4^ Although they were first described in humans in 1779 affecting a 16‐year‐old boy, trichobezoars are a phenomenon which almost exclusively affects females in the second and third decades of life and are quite uncommon in the pediatric age‐group.^5^, ^6^ The development of trichobezoars is strongly associated with trichotillomania (hair pulling) and trichophagia (hair eating), but there are reported cases of patients ingesting hair which is not their own (for example, that of other family members and even toy dolls).^7^ Presenting symptoms include abdominal pain, the presence of a palpable mass, nausea, vomiting, weight loss, constipation, and sometimes hematemesis. In cases where there is associated visceral perforation and peritonitis, mortality approaches 100% if operative intervention is not undertaken.^8^

## 2 CASE PRESENTATION

A previously healthy 7‐year‐old girl presented to a regional hospital in the middle of the night with a 3‐day history of colicky central abdominal pain associated with intractable vomiting. The pain had begun gradually in the central abdomen with eventual radiation across both the left and right sides. It had worsened over the next three days but was not exacerbated by movement or eating. Her bowels had opened normally on the day of hospital presentation, and there was no history of diarrhea, rectal bleeding, or urinary symptoms.

Other than mild asthma for which she took a preventative inhaler, there was no relevant past medical or psychiatric history. Her immunization status was up‐to‐date. There was no relevant family history of medical or psychiatric conditions. The patient had achieved all normal developmental milestones for her age and was of normal to above‐average intelligence. She performed well at school and lived with her parents and four siblings, all of whom were healthy. Her family came from a middle‐class background with no suggestion of social disadvantage or neglect.

On general inspection, the patient appeared pale and listless. Although she was febrile to 38.1°C, all other vital signs were normal for a child of her age (heart rate 120 beats/min, respiratory rate 20 breaths/min, blood pressure 115/65 mmHg, oxygen saturations 100% on room air, capillary refill <2 s). She appeared well nourished and weighed 24.1 kg, which put her between the 50th and 75th percentiles for her age.^9^ Her abdomen was soft but there was mild tenderness centrally and in the right iliac fossa without peritonism or rebound tenderness. A semimobile sausage‐shaped mass was palpable in the central abdomen.

**Figure 1:**
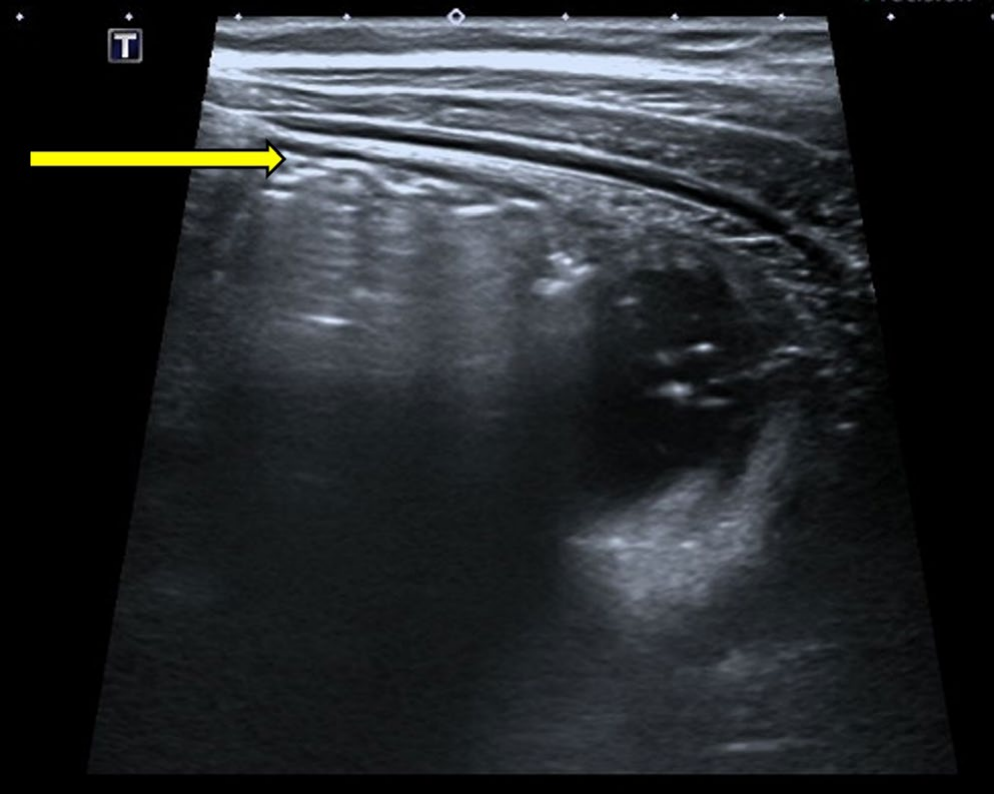
Abdominal ultrasound showing the presence of a mass associated with a superficial hyperechoic curvilinear structure (arrow) with posterior acoustic shadowing. This most likely represents a strand of hair within the trichobezoar

**Figure 2:**
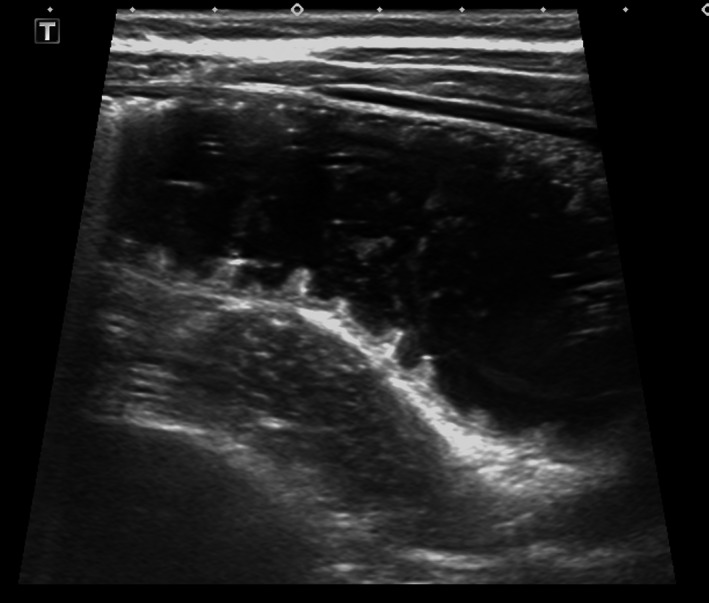
Ultrasound demonstrating distended and obstructed loop of proximal small bowel filled with heterogenous fecalized material, in keeping with acute obstruction

**Figure 3:**
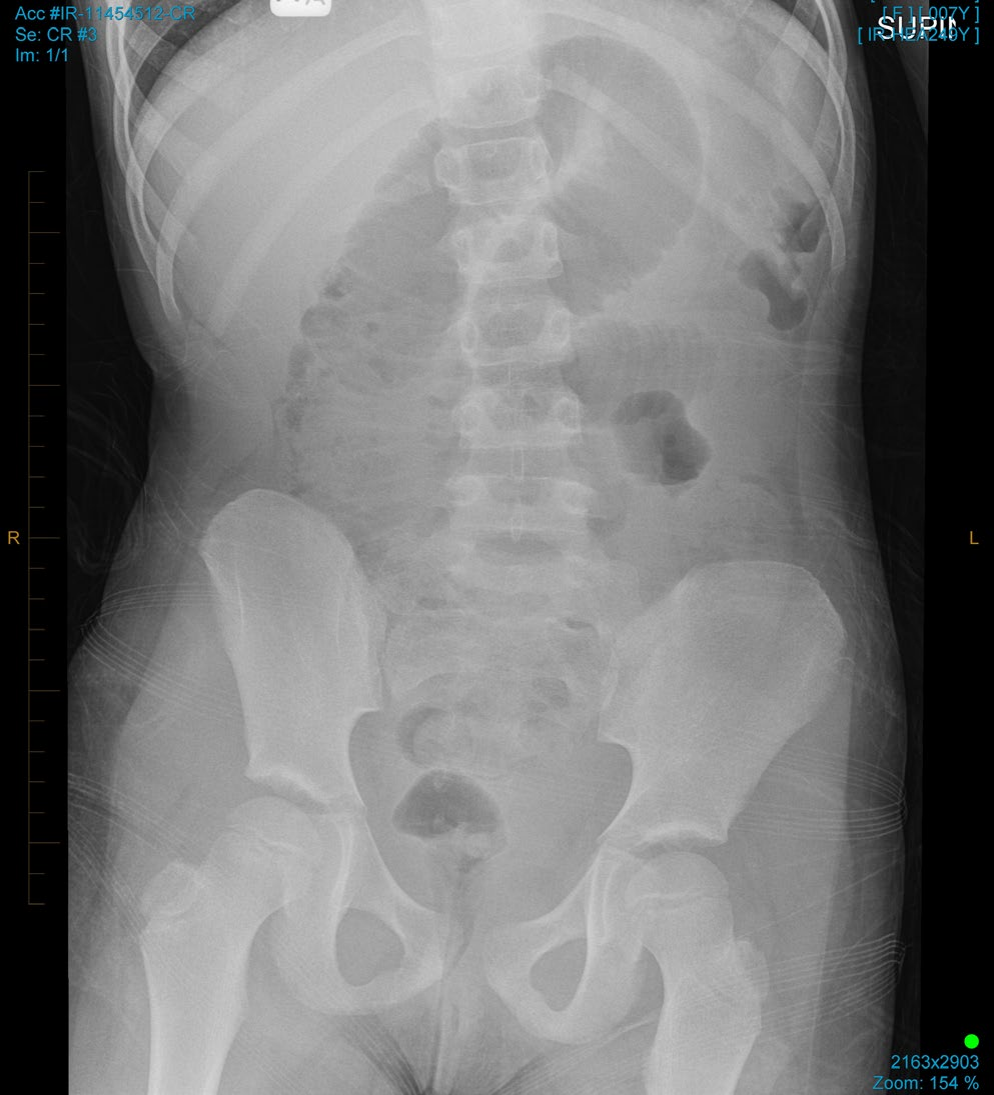
Plain abdominal X‐ray showing a distended loop of small bowel in the central abdomen, consistent with small bowel obstruction

Biochemical analysis revealed a white cell count of 25.0 × 10^9^/L (neutrophils 20.7 × 10^9^/L) and C‐reactive protein of 4 mg/L. Hemoglobin was 169 g/L, and renal function was normal. Urinalysis was also normal. Transabdominal ultrasound revealed an echogenic mass in the right iliac fossa which contained a hyperechoic curvilinear structure at its superficial margin associated with posterior acoustic shadowing (Figure 1). The appendix was not visualized and so acute appendicitis could not be excluded. There was no evidence of small bowel intussusception but a large amount of free fluid was noted within the abdomen and pelvis. Some distended loops of small bowel were also seen (Figure 2). Plain X‐ray was sought for further clarification, and it demonstrated a grossly dilated small bowel loop in the central and right abdomen consistent with a small bowel obstruction (Figure 3). A computed tomography (CT) scan of the abdomen and pelvis was considered but was not readily available in the middle of the night in our small regional hospital.

**Figure 4:**
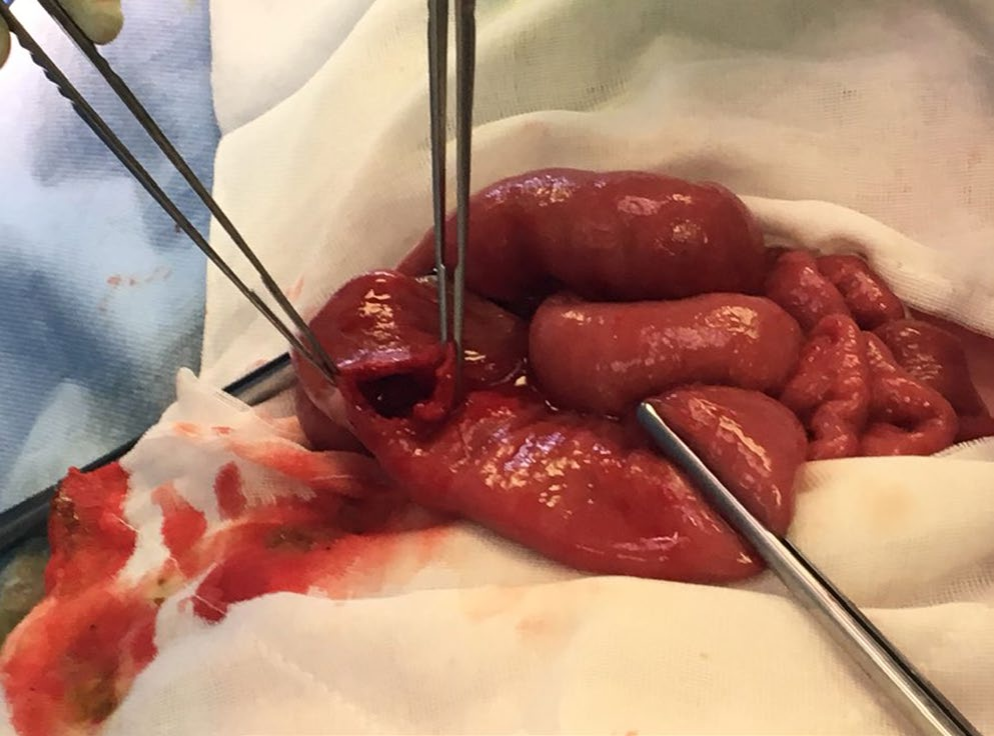
Intraoperative photograph demonstrating both distended proximal small bowel (left) and collapsed distal small bowel (right), consistent with bowel obstruction. A longitudinal enterotomy has been made on the antimesenteric border of the small bowel to facilitate retrieval of the trichobezoar

**Figure 5:**
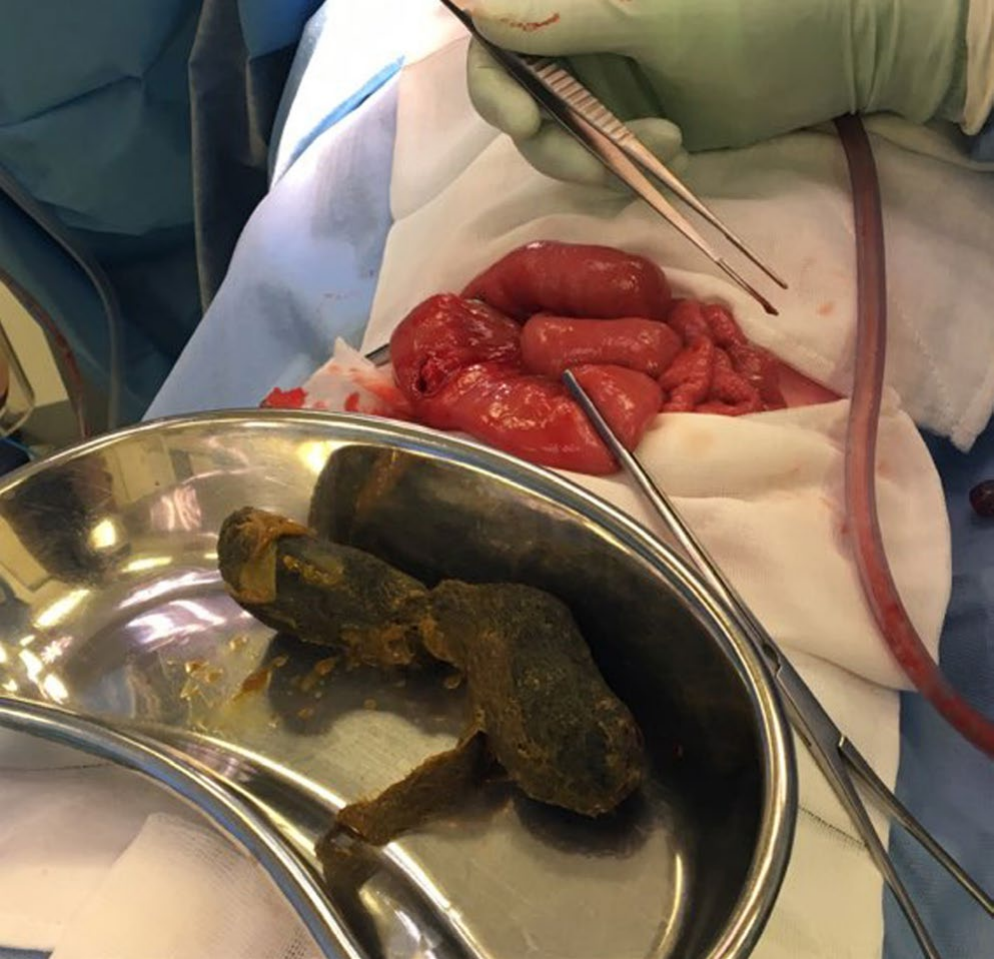
Photograph comparing the relative size of the retrieved trichobezoar with the small bowel in which it was impacted

**Figure 6:**
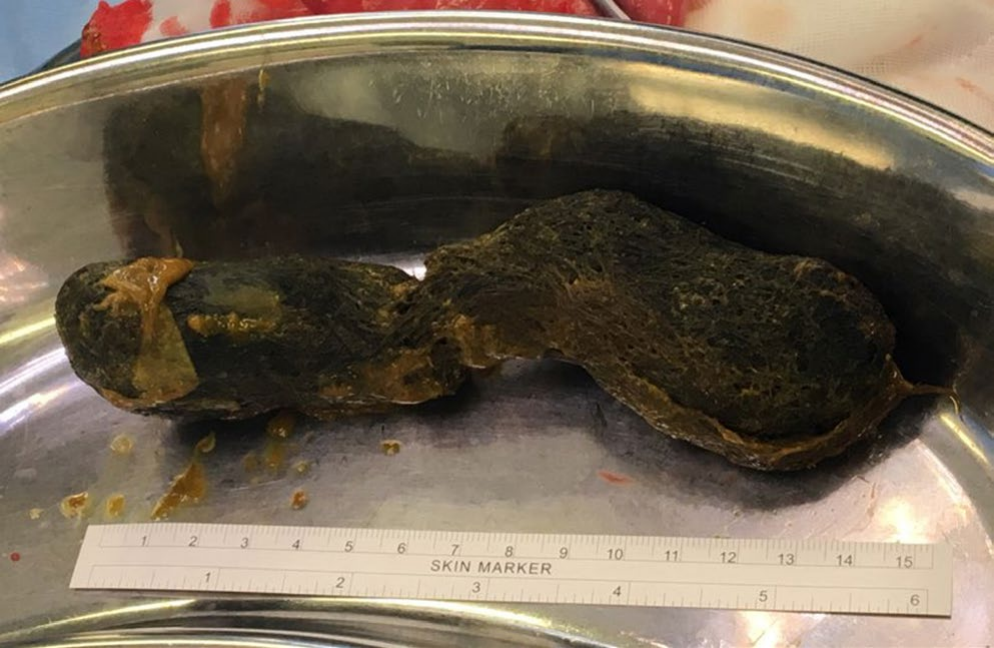
Close‐up photograph of trichobezoar demonstrating its size and shape corresponding to the outline of a child's stomach

The decision was made to bring the patient to theater for a diagnostic laparoscopy due to her clinical appearance, the presence of a palpable mass, fever, elevated neutrophil count, and abnormal imaging studies. Upon entry to the abdomen, markedly dilated proximal small bowel and copious serous free fluid was noted. The appendix appeared macroscopically normal, and the distal small bowel was collapsed. The small bowel was traced in a retrograde fashion from the ileocaecal junction until a firm, obstructing intraluminal mass was encountered in the proximal small bowel, approximately 60 cm from the duodenojejunal flexure. A 4 cm midline laparotomy was performed and the small bowel pulled through the wound after insertion of a small Alexis® wound retractor (Applied Medical). A 10 mm longitudinal enterotomy was made on the antimesenteric border just proximal to the intraluminal mass (Figure 4) and a large clump of impacted hair was retrieved, measuring approximately 15 × 5 × 3 cm. The shape of the mass was roughly cylindrical and conformed to the shape of the patient's stomach (Figures 5, 6). The enterotomy was repaired transversely with simple interrupted sutures and the abdominal wound closed in the usual fashion. Pathological examination confirmed the mass weighed 96 g and consisted of hair fragments and entrapped food particles, consistent with the diagnosis of trichobezoar.

Upon closer questioning of the patient's parents postoperatively, it was revealed that she had suffered from trichotillomania and trichophagia at the age of 4 years. It had always seemed to occur in response to physical fatigue, for example, at the end of a long day at preschool. This behavior had resulted in bald patches developing on her scalp which necessitated her parents shaving her head to prevent its continuation. After approximately 12 months, there was no objective ongoing evidence of this behavior and it was presumed to have ceased spontaneously. As a result, professional help had not been sought at the time. Both parents denied the existence of recent social stressors and had not observed any recent unusual behavior from their daughter. They seemed to be genuinely loving parents, and there was no indication of family dysfunction based on the observations of the treating surgeon.

The patient has an uneventful recovery and was discharged on the 5th postoperative day. She is planned to follow‐up in 4 weeks with the treating surgical team for routine review. Referral was also made to the childhood and adolescent mental health service for ongoing counseling regarding the underlying behavioral issues which resulted in trichotillomania and trichophagia, as well as to ensure cessation of this unwanted behavior.

## 3 DISCUSSION

Trichotillomania (TTM) was first described in 1889 and affects 1 in 2000 people in the general population. It involves the pulling of hair to the point of alopecia and is most commonly performed on the scalp, although eyelashes, eyebrows, and the axilla are all potentially susceptible.^7^ Only 30% of these patients will also engage in trichophagia and of those that do, only 1% will eventually develop a trichobezoar that requires surgical extraction.^7^ Only 14% of cases of TTM present before the age of 7 years but the condition is more likely to be benign and self‐limiting if onset is early. When disease onset occurs during adolescence or adulthood, there is a higher likelihood of it being chronic and associated with other psychiatric disorders such as anxiety, depression, and obsessive‐compulsive disorder.^7^

When trichophagia is repeated and long‐standing, the accumulated strands of hair are retained in the gastric rugae because there is insufficient friction for them to be moved forward by peristalsis.^5^ The smooth and slippery surface of the trichobezoar, aided by entrapped food particles and mucus, further impairs its passage through the pylorus.^3^ At least 10% of patients have a history of psychiatric disturbance, behavioral disorders, or mental retardation,^10^, ^11^ and a significant proportion of cases in children were observed in association with the recent death of a family member, recent parental separation, abuse, or socioeconomic disadvantage.^1^, ^7^, ^12^, ^13^

The majority of trichobezoars present with abdominal pain and a palpable mass and as previously discussed they are usually confined to the stomach.^14^ Occasionally there can be extension of a long “tail” of hair into the small intestine: This constitutes the so‐called Rapunzel syndrome, first described in 1968 and named after the German fairy‐tale character.^4^ There are a number of complications that can arise from trichobezoars depending on their size, location, and chronicity. Severe malnutrition (from vitamin and protein malabsorption) has been described, as have appendicitis, obstructive jaundice, and small bowel intussusception.^15^, ^16^, ^17^ Anemia can arise from both iron malabsorption and chronic ulceration, while acute hematemesis is present in up to 6% of cases.^4^ If left undiagnosed, acute bowel obstruction occurs in 26% of patients and peritonitis from visceral perforation will affect 18%.^18^

Once a trichobezoar is diagnosed, the primary management involves its retrieval. Surgery is the usual modality of choice with laparotomy being the most commonly performed procedure and is associated with a 99% success rate.^2^ Because these patients often present very late and with a large intraluminal mass, a totally laparoscopic approach may not be feasible in most cases, especially in children and small adults where the laparoscopic working space is limited by the patient's size. Nevertheless, the successful laparoscopic management of a large gastric trichobezoar in a 12‐year‐old girl has been described by Bustos et al,^19^ who managed to retrieve it and then repair the resultant gastrotomy via a minimally invasive approach. This is somewhat more challenging with a small bowel trichobezoar that has caused obstruction and dilatation of a significant length of small intestine. Open surgery therefore remains the current mainstay of treatment for this condition. Endoscopic retrieval has been described but is only successful in around 5% of cases of gastric trichobezoar, although it is slightly more effective for phytobezoar and lactobezoar.^2^, ^11^ Gastric lavage and dissolution therapy using lipase, cola, and papain have also been attempted but the size and density of trichobezoars have limited the success of these methods.^4^, ^8^, ^16^

A critical aspect of management which cannot be overlooked is secondary prevention. Recurrent trichobezoar has been reported and usually occurs in patients who have not had adequate psychiatric evaluation or follow‐up.^20^ Due to the rarity of this condition, there are no consensus guidelines on the long‐term management of TTM or for patients after trichobezoar extraction. No benefit has been demonstrated for the use of psychotherapy, fluoxetine, or clomipramine in the treatment of TTM although cognitive‐behavioral therapy (CBT) was associated with significant improvements over both clomipramine and placebo.^7^ The psychological underpinnings of TTM and trichophagia are the driving force which result in the physical manifestations of this disease and failure to address the root cause will predispose the patient to recurrent trichobezoars in the future. The importance of long‐term counseling and ongoing psychological evaluation cannot be underestimated.

Given the rarity of TTM and trichophagia, it is estimated that only 1‐2 people per million will be affected by trichobezoar. To our knowledge, there are only six documented cases of children younger than our patient having a trichobezoar^5^, ^17^, ^18^, ^21^, ^22^, ^23^ and in only one of these was the site of impaction in the small intestine.^23^ Rapunzel syndrome has been described as an uncommon form of this condition, but even more rare is the complete passage of a gastric trichobezoar through the pylorus into the small bowel causing a subsequent acute small bowel obstruction, as was found in our case. Our patient therefore represents a highly unusual variant of trichobezoar, given her age and the location of the pathology. In addition, the fact that she was of above‐average intelligence, performed well at school, and had no known history of parental neglect, recent social stressors, or mental retardation makes this diagnosis even more unexpected. To our knowledge, it is only the second documented case of pediatric trichobezoar in Australia, with the majority of previous reports originating from Asia or Europe.

## 4 CONCLUSION

Small bowel trichobezoar causing acute small bowel obstruction in a young child is an exceedingly rare condition that has only been described in the literature several times before. It should be suspected in young children who present with abdominal pain, a palpable mass, and signs of acute small bowel obstruction, especially when a history of trichotillomania is present. When this history is absent or not forthcoming, a very high index of suspicion is required to make the diagnosis clinically. Although its surgical treatment is relatively straightforward, the rarity and late presentation of this condition preclude the development of new techniques and approaches. As such, laparotomy remains the mainstay of treatment even in pediatric populations. Secondary prevention is also crucial and requires comprehensive psychiatric evaluation and follow‐up.

## CONFLICT OF INTEREST

The author declares no conflict of interest.

## AUTHOR CONTRIBUTION

AMFK: was the treating surgeon, performed the literature review, and composed the manuscript.
